# A multilevel analysis of the role personality play between work organization conditions and psychological distress

**DOI:** 10.1186/s40359-021-00703-6

**Published:** 2021-12-23

**Authors:** Annick Parent-Lamarche, Alain Marchand, Sabine Saade

**Affiliations:** 1 Department of Human Resources Management, Université du Québec À Trois-Rivières, 3225, Albert-Tessier, Trois-Rivières, QC G9A 5A7 Canada; 2 School of Industrial Relations, University of Montreal, C.P. 6128, Succ. Centre-ville, Montreal, QC H3C 3J7 Canada; 3 Department of Psychology, American University of Beirut, Jesup Hall, 102, Beirut, Lebanon

**Keywords:** Work organization conditions, Psychological distress, Workers, Personality traits, Agreeableness, Multilevel analysis, Moderation

## Abstract

**Background:**

Psychological distress in the workplace is usually attributed to work-related variables as well as non-work-related variables. Individuals working in the same organization can differ in terms of their appraisal of work-related stressors and coping strategies used to face them. The present study aims to evaluate the moderating role personality plays between work organizations conditions and psychological distress in a large sample of Canadian participants working in various occupations and workplaces.

**Methods:**

Multilevel regression analyses were conducted on a sample that followed a hierarchical structure with workers (N1 = 1958) nested in workplaces (N2 = 63). The direct contribution of workplace and personality was tested in a variance component model as a first step. Following this initial step, we introduced interaction variables by blocks of 11. Those interaction variables refer to each interaction combined with a specific personality variable.

**Results:**

Psychological demands, number of hours worked, job insecurity, neuroticism, and agreeableness were associated with higher levels of psychological distress. Inversely, decision authority, job recognition, self-esteem, locus of control were associated with lower levels of psychological distress. Lastly, agreeableness played a moderating role between low social support garnered from one’s supervisor and psychological distress.

**Conclusions:**

To intervene on work-related variables, organizations could reduce psychological demands, minimize the number of hours worked through job redesign, allow teleworking and encourage work schedule flexibility. To reduce job insecurity, organizations could explicitly communicate future organizational plans. In the same vein, decision authority could be targeted by reducing hierarchical steps and increasing autonomy. Lastly, the results pertaining to agreeableness stand in contrast with those of previous studies. We assumed that workers scoring high on agreeableness tend to put themselves last and please others first. These tendencies could make them more susceptible to health issues. With that said, work environments still need workers who are agreeable and nice to be around. To prevent high levels of agreeableness leading to psychological distress, training and information workshops are recommended. Those include stress management interventions and workshops pertaining to time management and relaxation techniques.

## Background

Psychological health should be of concern to organizations and society alike. In Canada, mental health problems affect about one in five individuals and two in nine workers [1]. Between 5 and 27% of the general population is believed to suffer from psychological distress [2]. In the US, 83% of employees report feeling stressed at work [3] while in Europe, 50% of workers experience stress regularly [4]. The associated costs of these mental health issues are conservatively estimated at $42.3 billion in direct costs and $6.3 billion in indirect costs [5]. On an organizational level, U.S. businesses lose up to $300 billion yearly due to occupational stress [3]. This same cost was estimated at £26 billion in the U.K. and at €617 in Europe [6].

Psychological distress in the workplace is usually attributed to work-related variables (i.e. physical and psychological demands, irregular schedules, and workplace harassment) and non work-related variables (i.e. family structure, support available from social networks outside of work) [7]. Even though work-related variables could be stressful, their effect does not seem to uniformly affect all workers [8]. Individuals working in the same organization could differ in terms of their appraisal of work demands and in coping strategies used to face them. Based on those individual differences, high job demands may not necessarily result in job strain for all workers [9]. For instance, conscientiousness [10–12] and neuroticism [13, 14] are both important personality traits. Additionally, agreeableness, conscientiousness, openness and emotional stability (also termed high neuroticism) moderated the negative impact of a high workload [15]. A recent study identified that the interaction between openness to experience and balanced contracts was significantly related to job satisfaction [16]. Neuroticism and agreeableness seem to play a moderating role between job strain and stress [17]. These personality traits have been shown to affect how individuals react to work-related stressors. Considering workers’ personality traits as well as personality-relevant constructs when evaluating the impact of work organization conditions on psychological distress is therefore important. Few studies examined the moderating role personality could play between work organization conditions and psychological distress [18–20]. That said, some recent studies have examined personality traits’ moderating role between work organization conditions and depression [21, 22] and between work organization conditions and burnout [23]. Based on the perceived gap in the literature, this study evaluates personality’s traits (i.e., Big Five) and personality-relevant constructs (i.e., self-esteem and locus of control) moderating role between work organization conditions and psychological distress. The study was conducted on a sample of 1958 workers in 63 companies.

## Empirical background

### Psychological distress

Most empirical studies evaluating mental health issues examined psychological distress, depression or burnout. Among the disorders referred to as mental health issues, psychological distress is worth considering. Compared to depression and burnout, psychological distress has a more global scope. Indeed, psychological distress refers to a pre-pathological state that is characterized by somatic, depressive and anxiety symptoms. In fact, this pre-pathological symptomatology largely corresponds to the definitions advanced by [24]. According to [25], taking into account the possible evolution of psychological distress over time is important. Symptoms such as fatigue, anxiety, irritability, depressive symptoms and somatic problems usually presented by an individual with psychological distress could lead to clinical depression, anxiety and burnout [26]. In addition to the aforementioned symptoms, a decrease in intellectual capacity (memory and concentration), an increase in aggressiveness, irritability, lack of energy, sleep problems, absenteeism, withdrawal, cognitive problems, excessive consumption of alcohol, drugs or medication could also be observed [7]. Among those health problems is psychosomatic illnesses and high blood pressure [7]. Over time, psychological distress could even increase the risk of premature mortality, suicide, and cardiovascular disease [7]. A recent study found work-related stress (although different from psychological distress) to be associated with early autonomic dysfunction of the cardiovascular system [27]. Understanding the determinants of psychological distress therefore seems essential to slow down the aggravation of symptoms and avoid its serious repercussions. Taking action at the outset of this negative chain of events therefore appears to be a privileged avenue for practitioners and stakeholders.

### Work organization conditions

Adverse psychosocial work variables/work organization conditions are now recognized as a significant source of psychological distress in the Western world. As for the Non-Western world, a recent cross-cultural study conducted in China and Cabo Verde found occupational stress to be associated with a higher risk of poor mental health [28]. Those psychosocial work variables can be categorized in four distinct dimensions: Task design, work demands, social relations and gratifications [29]. Those dimensions are similar to the ones advanced by popular stress at work models including the Job demands-control model [30]; the Job demands-control-support model [31]; the Effort-reward imbalance model [32] as well as the Demands-resources model [33].

#### Task design

*Decision Latitude* refers to the extent to which a worker can affect change in terms of her/his own work, her/his group work and the company’s policies [34]. Decision latitude comprises two main dimensions: Skill utilization and decision authority*.* On the one hand, *Skill Utilisation* refers to the possibility of using one’s skills and qualifications while having the possibility to harness new ones. On the other hand, *Decision Authority* refers to tackling work tasks using certain procedures at one’s own pace. Those work-related dimensions have been previously examined in the scientific literature [34]. A high decision latitude has been found to be associated with a lower level of psychological distress [35, 36].

#### Work demands

*Physical Demands* refer to variables exerting a physical load on the worker. Those variables include demands a worker faces that might pose a health or safety risk to her/him. Those variables usually include vibrations, extreme temperatures such as hot or cold, toxic fumes, smoke, loudness and dust. One also needs to consider any production related variables that might exert a physical load on the worker. As for their contribution to workers’ mental health, one study conducted by [7] highlighted a positive association between physical demands at work and psychological distress [7]. In the same vein, Chinese miners working in the smelting unit exhibited some of the highest rates of occupational stress compared to those working in other operational units [37]. Concerning *psychological demands*, those are variables exerting a mental load on the worker. Those variables usually include the quantity of work and pace to do it while facing opposing demands [30, 31]. Similarly, a high level of psychological demands seems to be associated with a higher level of psychological distress [18, 35, 36, 38, 39]. Lastly, excessive workload experienced by nurses in Poland seems to be associated with more burnout symptoms [40].

#### Social relations

*Social support* at work is usually garnered from one’s colleagues and supervisors. The quality of social interactions one entertains at work contributes to one’s mental health [41]. Indeed, social support enjoyed at work serves several purposes. On the one hand, social interactions provide a worker with recognition and help in doing one’s job. On the other hand, those interactions provide her/him with pleasure and reward for exerting effort [42]. Previous studies have supported the link between social support and psychological distress. More specifically, social support from one’s supervisor’s and colleagues seem to be negatively associated with psychological distress [18, 36, 38, 39, 43, 44].

#### Gratifications

*Gratifications* in the form of recognition, valorization, personal motivation, identification in one’s job are also worth considering. Gratifications at work also refer to job advancement, career opportunities and job security. Several studies have identified a negative association between job security and psychological distress [18, 38, 45].

Psychological distress in the workplace is usually attributed to work-related variables [3, 16, 33, 34, 36, 40–42] and in some cases, personality traits [3, 17, 18, 40, 57, 58]. The extent to which personality traits and personality-relevant constructs might moderate the association between work-related variables and psychological distress remains poorly understood. Based on this perceived gap, this study aims to focus on the moderating role personality traits/constructs could play between work-related variables and psychological distress.

### Personality traits and personality-relevant constructs

Personality traits and personality-relevant constructs are related. These two concepts both refer to personal characteristics and a propensity to behave in a certain manner in a situation [46]. In terms of personality traits, the Big Five have garnered a large recognition and refer to the following traits: *Extraversion, Agreeableness, Neuroticism, Conscientiousness, and Openness*.

#### Extraversion

Among those personality traits, *Extraversion* is worth mentioning. This personality trait is based on several dimensions such as conviviality, excitement seeking and the propensity to experience pleasant emotions such as joy and pleasure. A person scoring high on extraversion is usually someone who is convivial, active, talkative, optimistic, playful and geared towards others [47]. Individuals scoring high on the extraversion dimension tend to view difficulties through a positive length. They are also more likely to use problem-solving strategies while relying on social support [48]. Unsurprisingly, this personality trait has been found to be negatively associated with depression in workers [21]. That said, the link between extraversion and psychological distress per se has yet to be established.

#### Agreeableness

*Agreeableness* is considered a facet of interpersonal behavior. According to [47], agreeableness six defining characteristics include trust, straightforwardness, altruism, compliance, modesty and tender mindedness. Highly agreeable people are inclined to be sympathetic to others, to believe that others are well-intentioned [49], to be naïve, sympathetic, indulgent and cooperative [47]. Even though those individuals tend to pursue harmonious social interactions, they are more likely to navigate them with care [50]. Unsurprisingly, this personality trait seems to be negatively associated with psychological distress [20].

#### Conscientiousness

Being meticulous, consistent, eager, assiduous, dependable, determined and ambitious are all facets of *Conscientiousness* [47]. A study conducted by [21] found that scoring high on this personality trait is negatively associated with depression. To date, no study identified a significant association between this personality trait and psychological distress.

#### Neuroticism

*Neuroticism* is another personality trait worth examining. Neuroticism refers to one’s inclination to experience negative emotions such as fear, agitation, lack of confidence, anxiety, touchiness, social anxiety, and high impulsivity [51]. Because of the negative nature of those emotions, individuals scoring high on neuroticism tend to rely on unsuccessful coping mechanisms [52, 53]. Workers scoring high on neuroticism are more likely to suffer from psychological distress [20, 54]. Work-related stressors (e.g. Work-family conflict) are also subsequently likely to impact their mental health [20].

#### Openness

*Openness* refers to being intellectually curious, keeping an open mind and a rich emotional life [55]. In terms of its association with psychological distress, openness does not seem to have much of an impact on workers’ mental state [19, 38].

Even though the Big Five personality traits have enjoyed empirical support [56], personality-relevant constructs also merit our attention given that they are more precise than personality traits and usually show more variation over one’s life span. Those personality-relevant constructs refer to internal attributes that help an individual come up with solutions when faced with a new situation. Those personality-relevant constructs could therefore have repercussions on one’s adjustment to new and diverse situations [57, 58].

#### Self-esteem

According to [59], *Self-Esteem* refers to an individual's overall positive evaluation of oneself. This individual perception usually translates into an individual’s own approval (higher self-esteem) or disapproval (lower self-esteem). With regards to psychological distress, self-esteem seems to be directly [43, 60, 61] and indirectly associated with it [43]. In terms of its indirect role, a previous study found self-esteem to play a moderating role between social support and psychological distress [18].

#### Locus of control

*Locus of control* refers to one’s perception of the level of control she/he has over events surrounding her/him. An individual having an internal locus of control is likely to view important life events as dependent on her/his actions, efforts or skills rather than luck (external locus of control) [62, 63]. Relatedly, a distinction can be made between locus (i.e. internal versus external), controllability (subject to volitional alteration as opposed to cannot be willfully changed) and stability (fixed versus variable) in terms of dimensions of causality [64, 65]. Locus refers to the self versus the environmental responsibility for an outcome, while stability refers to perceived fluctuation over time (constant over time versus shift from moment to moment) [65] or the duration of a cause [64]. Controllability and stability represent two different dimensions in attribution processes. Controllability refers to the extent of control one has over a specific event. Ability and effort can be considered internal causes of success while ease of the task or help obtained from others can be considered external causes of success [64]. Typically, ability represents an internal, uncontrollable, and stable possible cause of an event, while effort represents an internal, controllable, and unstable possible cause of an event [64]. Locus of control influences one 's emotional response to an event [65]. Internal locus of control has been found to be associated with a lower level of psychological distress [19, 38, 60, 66]. These personality-relevant constructs have also been found to play a moderating role by attenuating the effects of adverse work organization conditions on psychological distress [19].

Based on the scientific literature previously presented, a gap can be identified. The moderating role personality traits and personality-relevant constructs play between work organization conditions and psychological distress levels remains unclear. The present study therefore aims to fill this gap by examining this potential moderating role in a large sample of Canadian participants working in various occupations and workplaces.

## Theoretical model

For the purpose of this study, we relied on several theoretical models pertaining to psychological and social determinants of work stress. Those models include [60, 67] social stress theory, as well as the comprehensive and multilevel model advanced by [68]. All those models rely on the same premise: an individual facing a certain level of stress at work will likely draw from her/his capabilities. In doing so, she/he is likely to avoid exhausting her/his mental resources. A situation is considered stressful when the demands placed by a stressor surpass a worker’s capabilities; subsequently impacting her/his psychological well-being [69]. That said, two individuals facing the same stressor wont necessarily respond in the same manner [67]. An individual’s characteristics (e.g., personality traits and personality-relevant constructs) are likely to influence her/his perception of work organization conditions as stressful [70]. Personality therefore seem to play an important role in a worker’s appraisal of a stressful work event. More specifically, personality traits and personality-relevant constructs can either reduce or amplify the impact of stressors at work on one’s psychological health. Based on the previously presented findings, we hypothesized that personality could play a moderating role between work organization conditions and psychological distress. Based on this assumption, we formulated seven specific hypotheses pertaining to each personality trait/construct.

### Hypotheses

#### *Hypothesis 1*

Extraversion plays a moderating role between work organization conditions and psychological distress. Individuals scoring high on extraversion are more likely to use problem-solving strategies while relying on social support [44]. Those same individuals are likely to participate in social activities resulting in more positive emotions [62].

#### *Hypothesis 2*

Agreeableness plays a moderating role between work organization conditions and psychological distress. Highly agreeable people tend to be more friendly to others and to pursue harmonious social interactions. Those harmonious social interactions could be useful in the face of work-related stressors.

#### *Hypothesis 3*

Conscientiousness plays a moderating role between work organization conditions and psychological distress. Highly conscientious people tend to be, among other things, determined and ambitious. Conscientiousness could fuel the individual's energy helping them face adverse work organization conditions. Lastly, conscientiousness seems to be associated with active coping strategies [71, 72].

#### *Hypothesis 4*

Neuroticism plays a moderating role between work organization conditions and psychological distress. Neuroticism refers to one’s tendency to experience negative emotions and the use of unsuccessful coping mechanisms. As such, one could hypothesize that neuroticism could amplify the negative effect of work-related stressors on psychological distress.

#### *Hypothesis 5*

Openness plays a moderating role between work organization conditions and psychological distress. Being intellectually curious and keeping an open mind can be helpful. More specifically, openness could help one look for ways to cope with work-related stressors.

#### *Hypothesis 6*

Self-Esteem plays a moderating role between work organization conditions and psychological distress. An overall positive evaluation of oneself could give an individual the confidence needed to face adverse work organization conditions.

#### *Hypothesis 7*

Locus of Control plays a moderating role between work organization conditions and psychological distress. An individual facing work stressors perceives that he/she has more control over important life events, he/she is more likely to act and look for solutions. Therefore, believing that one can cope with a work stressor could help attenuate the perception of threat.

**Fig. 1:**
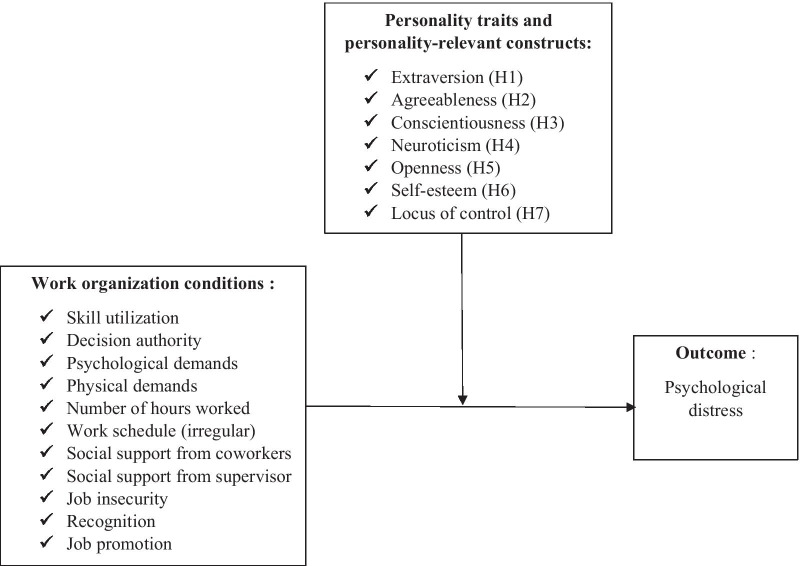
Theoretical framework

Based on the theoretical model previously presented, personality traits and personality-relevant constructs are presumed to influence an individual’s perception of work stressors by accentuating or attenuating their impact. High extraversion, agreeableness, conscientiousness, openness, self-esteem, internal locus of control and low neuroticism are likely to attenuate the negative impact of work-related stressors on psychological distress. The comprehensive theoretical framework we will test empirically is presented in Fig. 1.

## Method

### Participants

This project relied on a sample from the SALVEO study [68]. Ethics approval was obtained from several Universities (University of Montreal, McGill University, Laval University, Bishops University, and Concordia University). Data was collected from 63 Canadian companies. Workplaces were randomly selected from a more comprehensive list of 500 companies insured by a large insurance firm. This large insurance company was asked to refer clients that might be interested in participating in the study. Clients approached by the insurance company and interested in the study were referred to the research team. Participants were randomly selected from each company and asked to fill out a questionnaire individually. Those questionnaires were filled out during working hours on a touch screen computer brought by the researchers. Before participating in the study, the research team went over the consent form and made sure to explain confidentiality safeguards. After those steps had been taken, employees were asked to sign the consent form. No financial compensation was offered in exchange for participating in the study. As for employees who were absent at the time of our visit, an online version of the questionnaire was sent to them. In total, 2162 employees filled out the questionnaire with a response rate of 73.1%. Workplaces where data was collected were quite diverse in terms of services offered, products sold and markets served (e.g. engine manufacturing, software development, plumbing supplies, etc.). As for industry sectors, most companies worked in the tertiary sector (N = 44), some worked in the secondary one (N = 19) and others were part of a union. In terms of number of workers employed, those usually varied between 25 and 1900 employees (*M* = 247.1). After eliminating cases with missing values, a sample of 1958 employees remained. In terms of demographics distribution, less than half the sample (49%) were women with a mean age of 40.6 years old (SD = 10.8).

### Measures

#### Psychological distress

*Psychological Distress* was evaluated based on the twelve-item General Health Questionnaire (GHQ-12; e.g. Have you recently been able to enjoy your normal day-to-day activities?) and pertained to the recent past. Responses were measured on a four-point Likert-type scale (Less than usual/much more than usual). We derived a measure of psychological distress by summing the twelve items (α = 0.80) and using it as a continuous variable. The advantage of GHQ-12 is that it is short, and it can easily be scored. A score obtained on the GHQ-12 can range between 0 and 36, with higher scores indicating a more severe condition (i.e., higher levels of psychological distress) [73]. Most research to date has used the GHQ-12 to compute a global distress score [74]. Lastly, the GHQ-12 represents a valid and reliable measure of psychological distress in the general population in both French and English [2, 75].

#### Work organization conditions

The Job Content Questionnaire (JCQ) [76] was used to measure skill utilization, decision authority, psychological demands, and social support with a four-point Likert-type scale (Strongly disagree/strongly agree). *Skill Utilization* consisted of six items (e.g. My job requires a high level of skill; α = 0.80). *Decision Authority* comprised three items (e.g. I have a lot to say about what happens on my job; α = 0.79). *Psychological Demands* were assessed based on nine items (e.g. Waiting on work from other people or departments often slows me down on my job; α = 0.73). *Social Support from Colleagues* was measures based on four items (e.g., The people I work with take a personal interest in me; α = 0.83) while *Social Support* from one’s supervisor was evaluated based on four items (e.g., My supervisor pays attention to what I'm saying; α = 0.89). As for *Physical Demands,* we relied on the Effort-Reward Imbalance Questionnaire (ERI) [32]. This questionnaire allowed us to evaluate physical demands, career perspectives and job insecurity. Responses were also based on a four-point Likert scale (Strongly disagree/strongly agree). *Physical Demands* were evaluated based on a single item (e.g., My work requires physical effort). *Job Insecurity* was measured with two items (e.g., My employment security is poor; α = 0.65). *Job Recognition* comprised six items (e.g. I am treated unfairly at work, reverse coded; α = 0.82). *Job Promotion* included four items (e.g., My current occupational position adequately reflects my education and training; α = 0.69). *Number of Hours Worked* was obtained by summing hours worked per week in all jobs. *Irregular Work Schedule* was measured based on a single item evaluated on a four-point Likert scale (Never/all the time) and derived from the Quebec Health and Social Survey (QHSS-98).

#### Personality traits and personality-relevant constructs

The five general personality traits (Big Five) were measured based on the Mini International Personality Item Pool (Mini-IPIP; [77] and comprised 20 items. Although the Big Five and the Five-Factor model (FFM) (e.g., [47]) are not interchangeable, they largely overlap [78]. We drew from the International Personality Item Pool (IPIP; [79]) to identify several FFM personality traits [80]. Relatedly, the IPIP is based on the Big Five psychological factors. The suitability of the Mini-IPIP personality scale as a short measure of the FFM has been previously demonstrated in the scientific literature [80]. The items of this scale were distributed on a five-point Likert scale (Strongly disagree/strongly agree). *Openness* was evaluated based on four items (e.g. I see myself as someone who has difficulty understanding abstract ideas, reverse coded, α = 0.68). *Extraversion* was based on four items (e.g., I see myself as someone who doesn't talk a lot, reverse coded, α = 0.78). *Agreeableness* comprised four items (e.g. I see myself as someone who sympathizes with others’ feelings, α = 0.70), *Conscientiousness* four items (e.g. I see myself as someone who often forgets to put things back in their proper place, reverse coded α = 0.63), and *Neuroticism* four items (e.g. I see myself as someone who gets upset easily, α = 0.70). As for *Self-Esteem*, a personality-relevant construct, we relied on the Rosenberg Self-Esteem Scale with six items evaluated on a five-point scale (Strongly agree/strongly disagree; e.g. You are able to do things as well as most other people, α = 0.87). Lastly, we relied on a scale developed by [81] to measure *Internal Locus of Control*, a personality-relevant construct*.* This scale is based on seven items evaluated on a five-point additive scale (e.g. There is really no way you can solve some of the problems you have, α = 0.84).

#### Control variables

Our statistical analysis allowed us to account for the effect of variables associated with mental health issues at work. Adequate control has been made for variables that could confound the association between our independent variables and the outcome of interest. As such, our statistical model controlled for the following variables: *Sex, Age* [38, 44, 82–84], *Physical Activity* [85–87], *Marital Status* [84], *Parental Status* [44, 84], *Educational Level, Household Income* [88, 89], *Social Support* outside of the workplace [90], *Marital and Parental Tensions* [91], and *Stressful Childhood Life Events* [38].

*Sex* was coded as 0 = Man and 1 = Woman, and *Age* was coded in years. *Physical Activity* over the last 3 months was measured as the frequency of physical activities of 20 min or more. *Marital Status* was coded as 0 = Single, 1 = Living as a couple, and *Parental Status* as 0 = No, 1 = Yes. *Marital Tension* was based on four items evaluated on a binary-scale (Yes/No) [92] (e.g. You partner is not committed enough to your relationship, α = 0.70). *Parental Tension* was measured with three items on a two-point scale (Yes/No) [92] (e.g. A child's behaviour is a source of serious concern to you, α = 0.60). *Educational Level* was coded using the highest degree attained by the respondent on a ten category scale with ranks ordered according to the number of years needed to complete each degree from lowest to highest (1 = None, 2 = High school, 3 = Professional school, 4 = College (General), 5 = College (Technical), 6 = University (Undergraduate certificate), 7 = University (Bachelor’s degree), 8 = University (Graduate diploma), 9 = University (Master’s degree), 10 = University doctorate). *Household Income* was coded using pre-tax household income for the preceding 12 months on a twelve-category scale (1 = Less than $20 000, 12 = $120 000 or more). *Social Support* outside of the workplace was derived using four items with a two-point scale (Yes/No; e.g. Among family and friends, is there someone who would help you in time of need?). Lastly, *Stressful Childhood Events* (before the age of 18 years old) was measured using seven items with a two-points scale (Yes/no; e.g. Were you sent away from home because you did something wrong?) [92].

### Data analysis

Multilevel regression analyses [93, 94] were conducted with Stata 15 software. The data examined followed a hierarchical structure with workers (N1 = 1958) nested in workplaces (N2 = 63). In order to determine the contribution of workplace, personality and control variables on worker’s psychological distress, we included them in a variance component model. Note that before conducting our analyses, continuous predictors (including both independent and moderating variables) were centered around the mean. In other words, the mean was subtracted from each variable. By obtaining a mean of zero, our hope was to reduce multicollinearity [95, 96]. Following this adjustment, we introduced interaction variables by blocks of 11 (i.e., a single block at a time) with 29 additional variables (11 workplace variables; 7 personality variables; 11 control variables) included in each analysis. Those interaction variables refer to each of the interactions combined with a particular personality trait and a personality-relevant construct. The significance threshold used for the interactions was p ≤ 0.005 after Bonferroni’s correction. In order to reject the null hypothesis, we used a two-tailed probability established at p ≤ 0.05. This allowed us to determine the significance level of the combined variables as well as that of each individual regression coefficient. The random coefficients were examined based on halved p values [94].

## Results

Table 1 presents the descriptive statistics for the sample’s variables of interest along with the correlational analyzes. The results obtained indicated a low psychological distress score (*M* = 2.18, *SD* = 2.62).

**Table 1:** Descriptive and correlational statistics

	M	SD	1	2	3	4	5	6	7	8	9	10	11	12	13	14	15	16	17	18	19
1	2.18	2.62	1																		
2	17.72	3.40	− .20**	1																	
3	8.63	2.00	− .21**	.63**	1																
4	23.45	3.87	.17**	.20**	.06*	1															
5	1.99	0.96	.02	− .10**	− .12**	− .02	1														
6	40.39	9.09	.04	.11**	.10**	.15**	.04	1													
7	0.10	0.30	.06**	.03	.02	.16**	.12**	.13**	1												
8	12.54	1.95	− .19**	.27**	.24**	− .13**	− .11**	.00	− .06**	1											
9	11.94	2.60	− .18**	.32**	.36**	− .17**	− .15**	− .01	− .04	.37**	1										
10	3.78	1.30	.27**	− .18**	− .21**	.22**	.11**	− .01	.05*	− .27**	− .34**	1									
11	15.69	2.62	− .29**	.33**	.36**	− .24**	− .16**	− .01	− .07**	.51**	.62**	− .46**	1								
12	10.33	2.39	− .23**	.45**	.39**	− .10**	− .12**	.04	− .05*	.31**	.40**	− .32**	.51**	1							
13	19.40	3.44	− .34**	.30**	.27**	− .03**	− .04**	− .02	− .02	.25**	.18**	− .20**	.28**	.22**	1						
14	19.50	4.59	− .44**	.32**	.30**	− .10**	− .12**	.02**	− .06**	.29**	.27**	− .29**	.40**	.28**	.54*	1					
15	13.07	3.29	− .16**	.20**	.14**	− .01	− .03	.01	.07**	.20**	.11**	− .17**	.18**	.12**	.35**	.31**	1				
16	15.70	2.34	− .04	.09**	.05**	.03	− .08**	− .04	.01	.19**	.13**	− .10**	.16**	.10**	.26**	.21**	.40**	1			
17	10.54	2.93	.40**	− .20**	− .17**	.17**	.03	.01	.02	− .21**	− .18**	.22**	− .28**	− .23**	− .44**	− .46**	− .24**	− .17**	1		
18	15.02	2.48	− .13**	.06**	.06**	− .06*	− .02	− .03	− .04	.11**	.07**	− .14**	.14**	.07**	.25**	.24**	.09**	.23**	− .21**	1	
19	15.20	2.55	− .08**	.17**	.12**	.05*	− .04	.03	.05*	.08**	.07**	− .05*	.07**	.04	.29**	.24**	.25**	.21**	− .16**	.03	1

^a^ *p ≤ *0.05 (coefficients* ≥ 0.05) and **p ≤ *0.01(coefficients* ≥ *0.05)*

^b^ M = Mean; SD = Standard deviation; 1. = Psychological distress; 2. = Skill utilization; 3. = Decision authority; 4. = Psychological demands; 5. = Physical demands; 6. = Number of hours worked; 7. = Work schedule (irregular); 8. = Social support from coworkers; 9. = Social support from supervisor; 10. = Job insecurity; 11. = Recognition; 12. = Job promotion; 13. = Self-esteem; 14. = Locus of control; 15. = Extraversion; 16. = Agreeableness; 17. = Neuroticism; 18. = Conscientiousness; 19. = Openness

Model 1 presented in Table 2 indicates that psychological distress varies significantly between workplaces (σ_μ_^2^ = 0.072, p < 0.01), with a ρ = 0.01. In other words, 1% of psychological distress variance can be found between workplaces. Model 2 presents the main effect of workplace variables (skill utilization, decision authority, psychological demands, physical demands, number of hours worked, work schedule (irregular), social support from coworkers, social support from the supervisor, job insecurity, job recognition and job promotion) and that of personality traits and personality-related constructs (extraversion, agreeableness, neuroticism, conscientiousness, openness, self-esteem, locus of control) on psychological distress.

**Table 2:** Main effects of workplace and personality variables on psychological distress

	Model 1	Model 2
Fixed part		
Constant	2.18**	2.74**
Workplace		
Skill utilization		− .010
Decision authority		− .066*
Psychological demands		.048**
Physical demands		− .080
Number of hours worked		.012*
Work schedule (irregular)		.011
Social support from coworkers		.011
Social support from supervisor		.027
Job insecurity		.189**
Recognition		− .062*
Job promotion		− .003
Personality		
Self-esteem		− .058**
Locus of control		− .118**
Extraversion		− .000
Agreeableness		.081**
Neuroticism		.158**
Conscientiousness		.009
Openness		.017
Random part		
σ^2^ (companies)	0.072**	0.040*
σ^2^ (employees)	6.789**	4.662**
Fit		
X2	–	921.83
Df	–	(29)**

^a^*p ≤ .05 and **p ≤ *.01*

^b^The following variables were controlled for in Model 2: sex, age, educational level, household income, social support outside the workplace, stressful childhood events, marital status, parental status, marital stress, parental stress, physical activity. (Unstandardised coefficients)

The results obtained indicate a significant variation in the level of psychological distress across individuals/employees (σ_ε_^2^ = 4.662, p < 0.01) and across workplaces/companies (σ_μ_^2^ = 0.040, p < 0.05). *Decision Authority*, *Job Recognition*, *Self-Esteem*, and *Locus of Control* were negatively associated with levels of psychological distress. Inversely, *Psychological Demands*, *Number of Hours Worked*, *Job Insecurity*, *Agreeableness* and *Neuroticism* were positively associated with workers’ level of psychological distress. Finally, inspection of the fit indices showed that the model met the recommended criteria [94].

### Interaction results

**Fig. 2:**
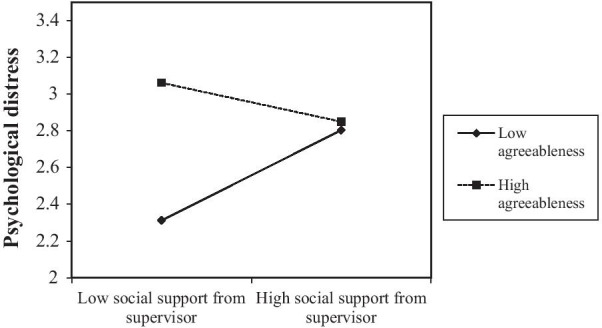
Agreeableness and social support from supervisor interaction. *Note*: Unstandardised coefficients

After applying a Bonferroni correction (p ≤ 0.005) to the 77 interaction tests, only agreeableness seemed to interact with social support from the supervisor (*X2* = 21.81; *Df* = 11; *p* ≤  0.05) in explaining workers’ level of psychological distress. As shown in Fig. 2, agreeableness (β = − 0.029; *p* ≤  0.005) played a moderating role between social support from the supervisor and psychological distress. Whe agreeableness level is high, low social support from one’s supervisor seems to increase the risk of psychological distress. Inversely, when agreeableness is low, inadequate social support from one’s supervisor seems to decrease the risk of psychological distress. Lastly, when social support from one’s supervisor is high, agreeableness does not seem to make a difference in terms of risk of suffering from psychological distress.

## Discussion

In this study, we examined the moderating role personality traits and personality-relevant constructs play between work-related stressors and psychological distress. Despite the absence of hypotheses to their effect, psychological demands, number of hours worked, and job insecurity were found to be associated with higher levels of psychological distress (direct effects). These results align with those of previous studies with regards to psychological demands [18, 35, 36, 38, 39], number of hours worked [97], and job insecurity [18, 38, 45]. Three work organization conditions seem to be the most harmful in terms of psychological distress. Inversely, decision authority and job recognition seem to play a protective role in terms of psychological distress. The results pertaining to decision authority are shared with those of previous studies [35, 36]. As for job recognition, one study was able to identify a negative association between job recognition and depressive symptoms [98]. Decision authority and job recognition seem to be the most effective in mitigating workers’ pre pathological symptoms. Surprisingly, social support garnered from one's colleagues and supervisor, both important job resources, was not associated with psychological distress. Our results do not align with those previous studies [18, 36, 38, 39, 43, 44]. More specifically, previous studies found social support to be negatively associated with psychological distress. Similarly, police officers with organizational support seem to display lower burnout levels [99]. Even though social support was found to be negatively correlated to stress levels in a recent study [100], we were not able to replicate this finding in our regression model. As for the direct effect of personality traits and personality-relevant constructs, our results indicate that self-esteem and internal locus of control are directly associated with a lower level of psychological distress. Inversely, agreeableness and neuroticism are directly associated with a higher level of psychological distress. Those results are consistent with those of previous studies on self-esteem [43, 60, 61], locus of control [19, 38, 60, 66] and neuroticism [20, 54].

The results pertaining to agreeableness stand in contrast with those of previous studies on workers [20] and individuals from the general population [101]. According to [50] agreeableness is a disposition that increases one’s motivation to establish and maintain positive relationships with others. Individuals scoring high on agreeableness usually engage in more altruistic behaviors at work [102, 103] therefore engaging in self-sacrifice [103]. Based on this premise, we hypothesized that empathic, sympathetic, nurturing, patient, and cooperative individuals are less likely to experience psychological distress because of their positive disposition. It is also possible that once the same individual reaches a certain point, agreeableness becomes a burden resulting in psychological distress. This stands in contrast to individuals scoring low on agreeableness who tend to be more self-focused and are therefore less likely to suffer from psychological disorders [104]. Agreeableness helps an individual be sensitive to victims in need and empathize with them [105] therefore drawing from one’s emotional resources. The same reasoning could be extended to the workplace. Workers scoring high on agreeableness tend to engage in more proactive behaviors such as helping others in order to maintain a good social relationship [106]. Workers’ desire to please others and maintain good social relationships could lead to difficulties with saying no. Those people-pleasing tendencies could subsequently lead to a higher risk of suffering from psychological distress. Helping others instead of avoiding difficult situations may therefore prove stressful over time.

Results obtained refute hypotheses pertaining to the significant role personality traits and personality-relevant constructs could play between work organization conditions and psychological distress (H1; H3; H4; H5; H6; H7). More specifically, extraversion, conscientiousness, neuroticism, openness, self-esteem, and internal locus of control did not moderate the association between work organization conditions/work-related stressors and psychological distress. We expected problem-solving strategies, the tendency to experience positive emotions, being intellectually curious, having a positive evaluation of oneself and having a perception of control over important life events to attenuate the negative impact of work-related stressors on psychological distress. Those same results do not align with stress theories [67–69]. Stress theories advance that an individual facing a certain level of stress at work will likely draw from her/his capabilities to face it. Further investigations should be made to verify if those personality traits and personality-relevant constructs could help with work-related stressors. Similarly, other individual characteristics should be tested in the future to revise and build on these theories. That said, one personality trait did play a moderating role between low social support from one’s supervisor and psychological distress. High agreeableness seems to accentuate the negative impact of low social support on workers’ mental health. Despite its significance, the finding obtained contradicts hypothesis (H2). H2 stipulates that agreeableness could attenuate the negative impact of work-related stressors on psychological distress. This surprising finding could be explained as follows: workers scoring high on agreeableness tend to put themselves last and tend to please others. Those tendencies make them more susceptible to health issues [107]. Given the perceived importance of cooperation, workers scoring high on agreeableness are more likely to rely on supervisor support to shield them from psychological distress.

### Practical implications

The results of this study indicate that five work organization conditions are associated with higher psychological distress levels. Based on those findings, we would advise organizations to reduce psychological demands and minimize the number of hours worked. Having more people tackle the same tasks, providing more time per person to accomplish the same tasks and reducing the number of tasks per person are possible options [108]. As for organizations with sufficient financial resources, job redesign, teleworking, flexibility in terms of work schedules and reduced working hours could be worth considering [109]. In the same vein, job insecurity could be minimized by increasing workers’ perceived control at work [110]. In pursuing those intervention targets, one should address: communication, participation, and employability. Inadequate communication about future events is a contributor to workers’ perceived insecurity [110]. Inversely, explicit communication about future organizational plans seems to effectively reduce insecurity [111–113]. Open and timely communication increases the predictability and perceived controllability of what is to come; reinforcing one’s perception of being valued and respected [110]. Another means of reducing job insecurity is by allowing workers to partake in the decision-making process related to the organization’s future [114]. Organizations can also take preventative steps by strengthening employees’ skills and eventually facilitating finding a new job [110]. Decision authority could be targeted by reducing hierarchical steps and increasing autonomy [108]. To do so, workers should be allowed to cultivate their creativity, take initiative, have some leeway in choosing their work methods and in controlling their work pace. Even tough we did not find an association between social support and psychological distress, improving supervisor and colleagues' support could be one way of managing work-related stress and workers' well-being (i.e., lower psychological distress). In light of some recent scientific findings, fostering a supportive work environment and encouraging job support could be a human resource strategy to reduce work-related stress [115, 116]. Finally, recognition problems could be addressed by means of psychosocial interventions. Among those interventions are those implemented in the context of the Quebec Healthy Enterprise [117]. Interventions, especially those targeting low rewards could successfully minimize workers’ psychological distress [117]. More specifically, recognition interventions in the “Management Practices” area could be implemented. Examples of such interventions include raising awareness, training managers to provide job recognition and providing attention and respect to the employees on a daily basis. Employees should also be encouraged to present novel ideas facilitating task execution and enhancing the work environment [118].

The results obtained from this study highlight the important role workers’ personality traits and personality-relevant constructs play in terms of their mental health. Based on those findings, self-esteem and locus of control could be targeted as they seem to be amenable to change [119]. Organizational psychologists could help companies intervene on those personality-relevant constructs by offering training and coaching. In terms of career mobility, human resource managers could rely on valid psychometric tests to ensure that workers being considered for promotions are psychologically ready. Those individuals should be armed with a strong self-esteem and an internal locus of control to help prevent psychological distress. In doing so, one needs to pay attention not to favor certain employees over others by and preserve employees’ perception of organizational justice. One way of selecting the right employee for the right job is by training potential candidates. As for neuroticism, this personality trait is considered hard to change as it tends to remain stable over time [54]. Strategies to avoid potential mental health problems include strengthening an individual’s coping strategies and implementing educational programs in the workplace [54].

Lastly, agreeableness seems to play a direct and moderating role between work organization conditions and psychological distress. The moderating effect pertains to agreeableness’ role between social supervisory support and psychological distress. Agreeableness is a general personality trait that tends to remain stable or decline late in life [56]. In fact, work environments need workers who are agreeable, cooperative, empathetic, altruistic, indulgent and nice to be around. High levels of this personality trait could still be concerning as those workers tend to engage in altruistic behaviors [103] that might extend to carrying the burden of those around them [120]. Priority management techniques and boundary setting should also be taught.

### Limitations and recommendations for future research

This study has limitations worth mentioning. Among those limitations, is the use of a cross-sectional design. This design prevents us from drawing causal relationships between the variables examined. The possibility of an inverse relationships is also worth considering. More specifically, it is possible that workers struggling with psychological distress are more likely to negatively rate their work organization conditions. Relying on secondary data based on the larger SALVEO study dictated the measures used and the variables evaluated. Considering other mental or personality disorders such as generalized anxiety disorder, sleep disorders, or avoidant personality disorder amongst others could have been interesting. In the same vein, examining the contribution of various work-related variables such as leadership style, diversity management practices, technostress at work, etc. could be pertinent. Relying on measures drawn from the same source could result in common variance bias. That said, the bias incurred using measures in the same context is minimised due to the sample diversity. Workers included in the study were drawn from 63 companies that were quite diverse in terms of company size, economic sector and employees’ unionization or lack of. A previous study conducted on the same data has confirmed that the common method bias should not be a source of concern as it remained small [68]. Similarly, low employee response rate (41%) could have resulted in selection bias. More specifically, companies with a large number of employees struggling with mental health issues are more likely to participate in this type of study. Since random regression coefficients were not tested in our study, whether associations were the same across workplaces remains unclear. Lastly, other variables related to the physical location of the workplace could have been pertinent. Those include dust, noise, cold, heat, etc., human resource policies, practices, health and safety and other work-related variables likely to be mentioned in an employment contract.

## Conclusions

In sum, this study is novel in that it evaluates the moderating role personality plays between work organization conditions and psychological distress. To the best of our knowledge, no previous study has attempted to evaluate the same research question using the same variables. Additionally, psychological distress represents a pre-pathological condition that could lead to more severe mental and physical health problems. Identifying the determinants of these mental health problems as early as possible could help circumvent some of those negative consequences. Therefore, these findings could be particularly useful for practitioners. Swift action can be taken at the outset of signs of psychological distress before developing into a more severe mental health problem (e.g., burnout and depression). These results obtained from this study also add the agreeableness literature. Agreeableness was found to accentuate the negative impact of work-related stressors on psychological distress. We assume that workers scoring high on agreeableness tend to put themselves last and please others first. Those tendencies are likely to make them more susceptible to health issues. As a take-home message, we wish to emphasize that work environments still need workers who are agreeable, cooperative, empathetic, altruistic, indulgent, and nice to be around. Training and information workshops for practitioners and stakeholders are recommended to prevent high levels of agreeableness leading to psychological distress. Those include stress management interventions and workshops pertaining to time management and relaxation techniques that could be beneficial for workers’ well-being. Future studies are needed to understand how personality mitigates or amplifies work-related stressors’ effect on psychological distress. Based on those findings, we recommend that future researchers expand their search for individual attributes while considering the intricacies of personality traits and personality-relevant constructs. In doing so, empathy, emotional intelligence and coping strategies are worth examining.

## Data Availability

The datasets analysed during the current study are not publicly available in order to respect the privacy of research participants, but are available from the corresponding author on reasonable request and with permission of Alain Marchand.

## Abbreviations

GHQ-12: General Health Questionnaire
JCQ: Job Content Questionnaire
ERI: Effort-Reward Imbalance Questionnaire
QHSS-98: Quebec Health and Social Survey;
IPIP: International Personality Item Pool
FFM: Five-Factor model
